# Supplementary material for: The relevance of gene flow with wild relatives in understanding the domestication process
Source: R Soc Open Sci. 2020 Apr 15;7(4):191545. doi: 10.1098/rsos.191545 (PMC7211868; doi:10.1098/rsos.191545)
Supplement: Supplementary Material [file rsos191545supp2.pdf]

**SUPPLEMENTARY MATERIAL**

Citation: Moreno-Letelier A, Aguirre- Liguori JA, Piñero D, Vázquez-Lobo A, Eguiarte LE. 2020 The relevance of gene flow with wild relatives in understanding the domestication process. R. Soc. Open Sci. 7: 191545. http://dx.doi.org/10.1098/rsos.191545

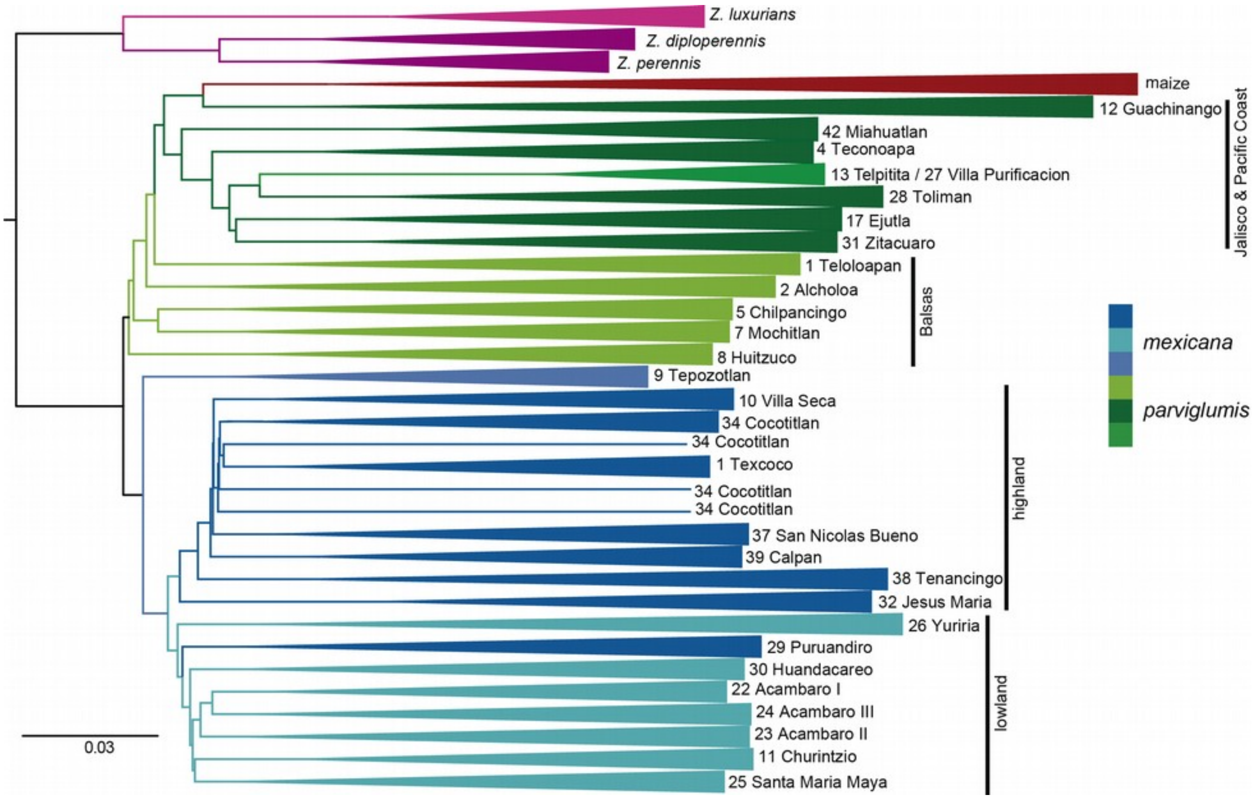


Fig. S1.- Identity by state genetic distance dendogram based on 30 673 SNPs, including three outgroups: *Zea luxurians, Zea perennis* and *Zea diploperennis.* Populations where all accessions clustered together were collapsed.

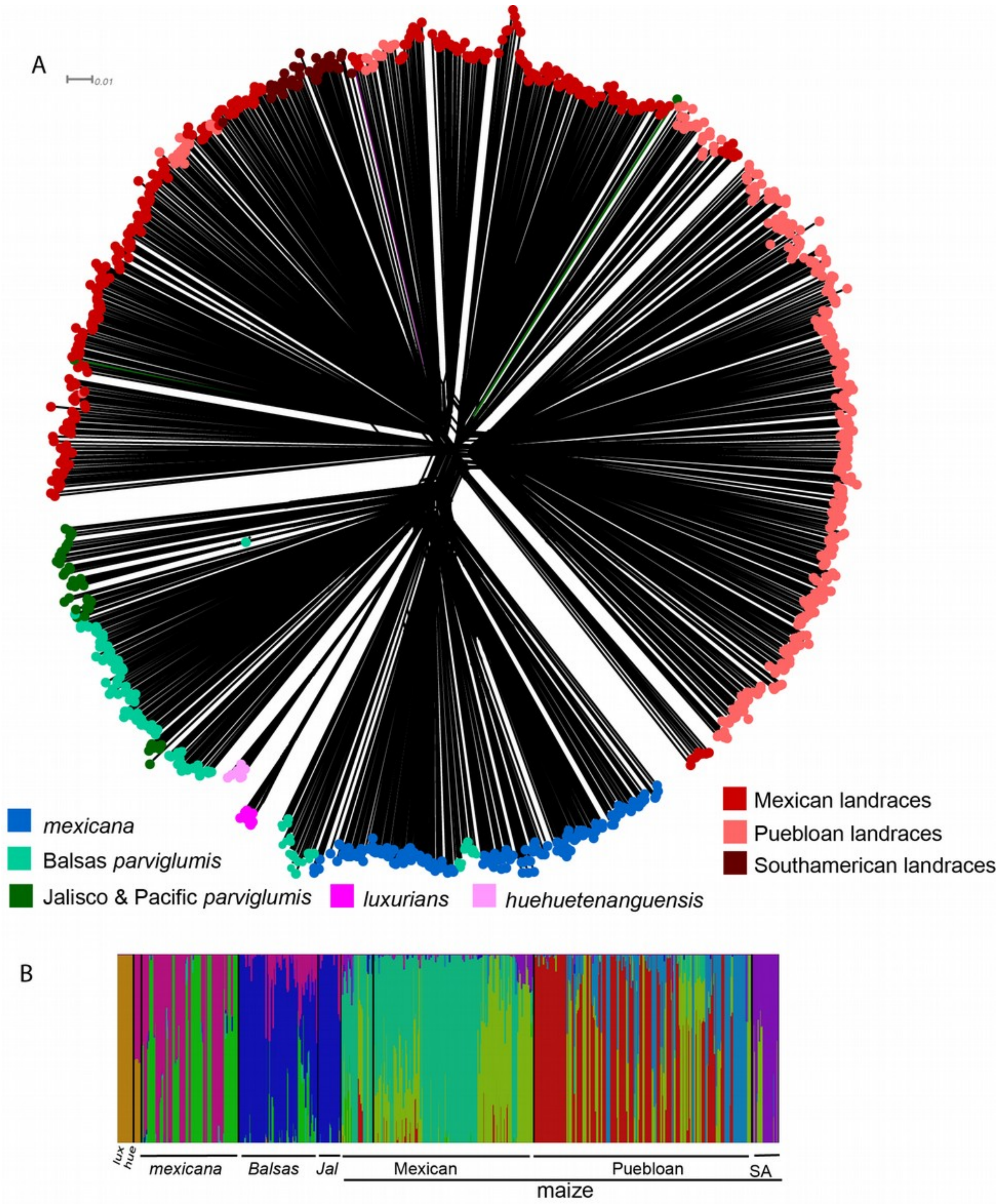


Fig. S2.- A*)* Identity by state genetic distance network with 122 085 SNPs from Swarts et al. (2017), and B) fastStructure analysis with K=9 of the same dataset. Most *parviglumis* samples come from the Balsas region, with only a few individuals from Jalisco. This figure confirm that *mexicana* is a heterogeneous groups, introgression between the Balsas Basin and *mexicana* and genetic differentiation between *parviglumis* and maize landraces.

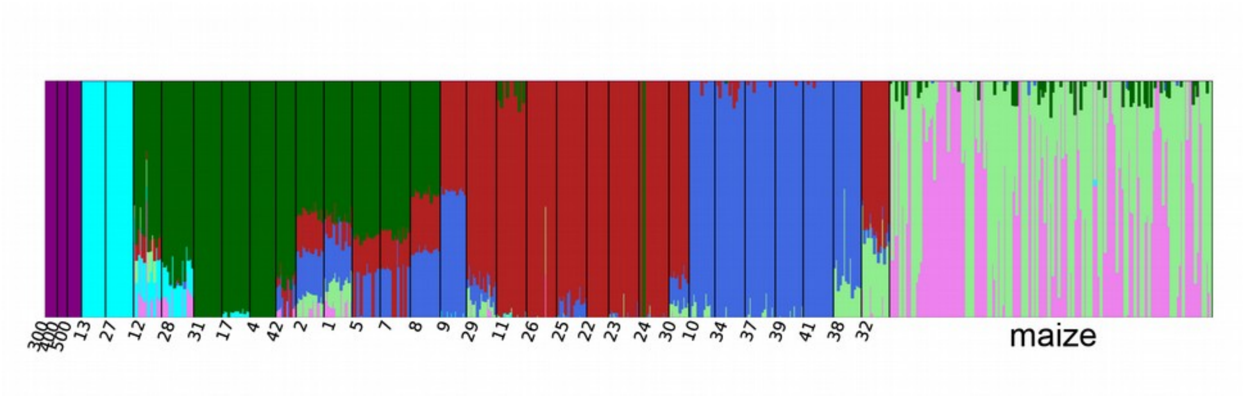


Fig. S3.- fastStructure analysis of the chip dataset, with K = 7.  The substructure detected occurs within maize.

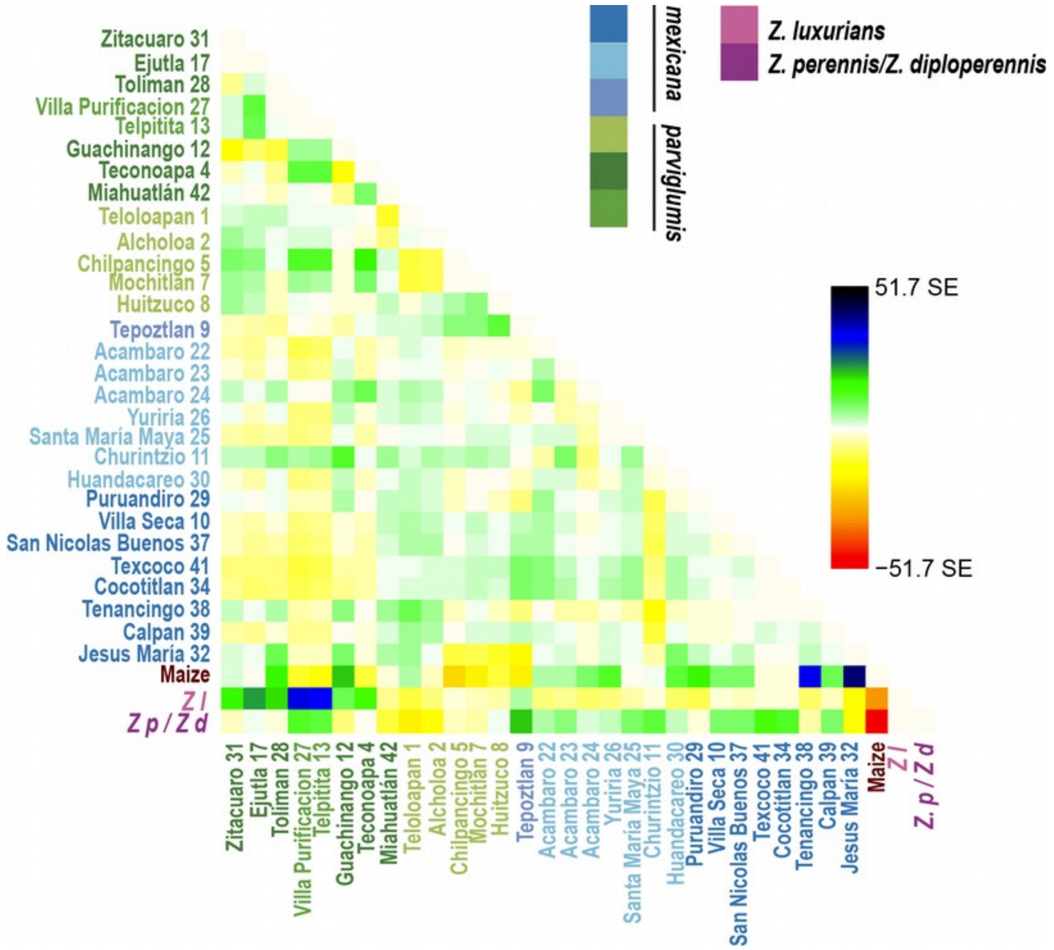


Fig. S4.- Genetic covariance matrix used to estimate the number of migration events to be modelled by Treemix 1.2. Blue colours indicate a higher covariance than expected based on the Maximum Likelihood population graph in Fig. 4.

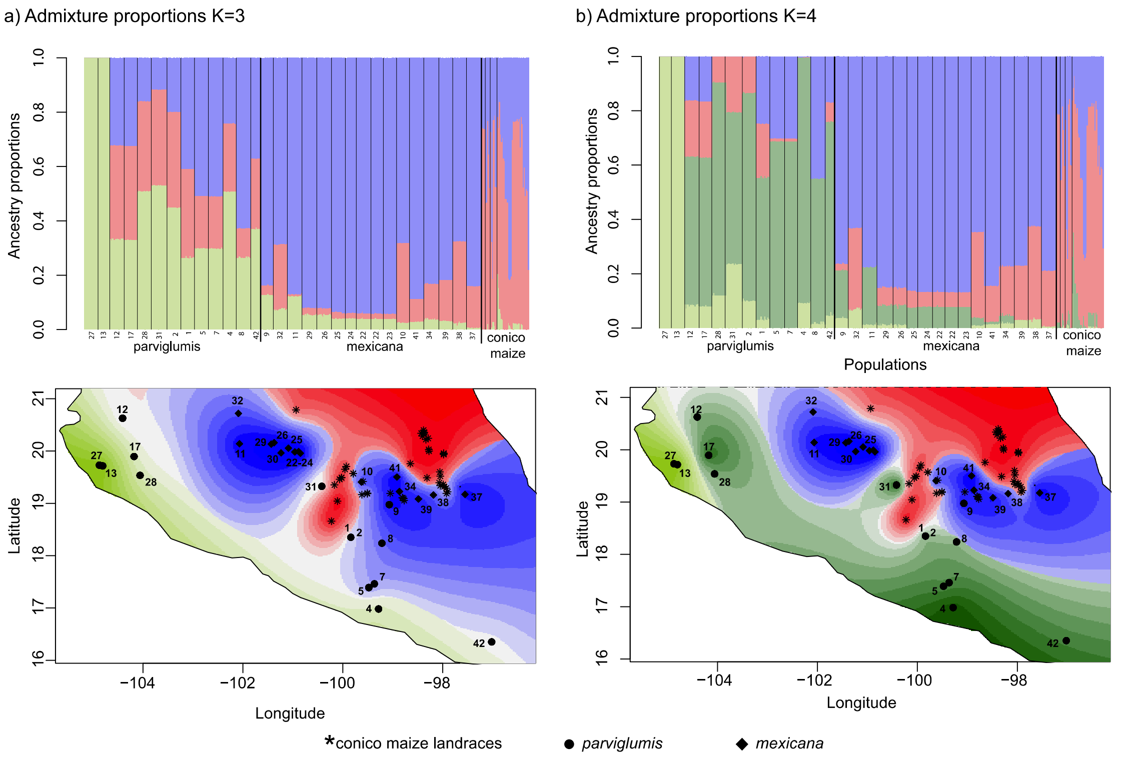


Fig. S5.- Admixture proportions on the geographic space of teosinte (*parviglumis* and *mexicana*) and conico maize landraces, sorted by longitude. Balsas populations (1, 2, 4, 7 and 8) show a high level of admixture with highland *mexicana.* Outgroups or non-conico maize landraces were not included in these analyses.

Table S1.- Locality information of teosinte populations used in this study.

| **Subspecies** | **Locality** | **State** | **Altitude** | **Long** | **Lat** | **Affinity** |
|---|---|---|---|---|---|---|
| Z. mays spp. parviglumis | Teloloapan | Guerrero | 1649 | -99.84108 | 18.34983 | Midland |
| Z. mays spp. parviglumis | Guachinango | Jalisco | 1426 | -104.4079 | 20.6273 | Midland |
| Z. mays spp. parviglumis | Telpitita | Jalisco | 504 | -104.8 | 19.715 | Lowland |
| Z. mays spp. parviglumis | Ejutla | Jalisco | 1317 | -104.17 | 19.896 | Midland |
| Z. mays spp. parviglumis | Alcholoa | Guerrero | 1439 | -104.407 | 20.627 | Midland |
| Z. mays spp. mexicana | Villa Seca | Mexico | 2581 | -99.627 | 19.407 | Highland |
| Z. mays spp. mexicana | Churintzio | Michoacán | 1846 | -102.068 | 20.139 | Midland |
| Z. mays spp. mexicana | AcámbaroI | Guanajuato | 1861 | -100.88 | 19.99 | Midland |
| Z. mays spp. mexicana | AcámbaroII | Guanajuato | 1878 | -100.95 | 19.98 | Midland |
| Z. mays spp. mexicana | Santa Ana Maya | Michoacán | 1849 | -101.08 | 20.05 | Midland |
| Z. mays spp. mexicana | Yuriria | Guanajuato | 1856 | -101.37 | 20.16 | Midland |
| Z. mays spp. parviglumis | Villa Purificación | Jalisco | 572 | -104.87 | 19.73 | Lowland |
| Z. mays spp. parviglumis | Toliman | Jalisco | 1369 | -104.058 | 19.53 | Midland |
| Z. mays spp. mexicana | Puruandiro | Michoacán | 2002 | -104.43 | 20.133 | Highland |
| Z. mays spp. mexicana | Huandacareo | Michoacán | 1844 | -101.23 | 19.96 | Midland |
| Z. mays spp. parviglumis | Zitacuaro | Michoacán | 1383 | -100.42 | 19.32 | Midland |
| Z. mays spp. mexicana | Jesus María | Jalisco | 2176 | -102.089 | 20.719 | Highland |
| Z. mays spp. mexicana | Cocotitlán | México | 2252 | -98.866 | 19.22 | Highland |
| Z. mays spp. mexicana | San Nicolás Buenos Aires | Puebla | 2375 | -97.55 | 19.17 | Highland |
| Z. mays spp. mexicana | Tenancingo | Tlaxcala | 2306 | -98.18 | 19.159 | Highland |
| Z. mays spp. mexicana | Calpan | Puebla | 2447 | -98.48 | 19.08 | Highland |
| Z. mays spp. parviglumis | Teconoapan | Guerrero | 581 | -99.28 | 16.98 | Lowland |
| Z. mays spp. mexicana | Texcoco | Mexico | 2234 | -98.91 | 19.5 | Highland |
| Z. mays spp. parviglumis | San Cristobal | Oaxaca | 982 | -97.02 | 16.35 | Lowland |
| Z. mays spp. parviglumis | Chilpancingo | Guerrero | 1201 | -99.477 | 17.39 | Midland |
| Z. mays spp. parviglumis | Mochitlán | Guerrero | 1107 | -99.36 | 17.45 | Midland |
| Z. mays spp. parviglumis | Huitzuco | Guerrero | 1101 | -99.218 | 18.23 | Midland |
| Z. mays spp. mexicana | Tepoztlán | Morelos | 1664 | -99.07 | 18.97 | Midland |

Table S2.- Maize landraces used in the study

| ID | Landrace | State | Altitude (m) | Longitude | Latitude |
|---|---|---|---|---|---|
| maiz_1 | Apachito | Chihuahua | 2106 | -108.0796 | 29.1131 |
| maiz_10 | conico | Chihuahua | 1734 | -108.0082 | 27.4348 |
| maiz_100 | blando de sonora | Sinaloa | 299 | -108.383 | 27.435 |
| maiz_101 | conico | Tlaxcala | 2557 | -98.0483 | 19.5189 |
| maiz_102 | conico | Hidalgo | 1698 | -98.3833 | 20.3975 |
| maiz_103 | conico | Mexico | 1779 | -100.1083 | 19.0439 |
| maiz_105 | MUSHITO | Chiapas | 1967 | -92.3078 | 15.3631 |
| maiz_106 | PEPITILLA | Baja California Sur | 5 | -111.9864 | 26.8905 |
| maiz_108 | dulcillo del noroeste | Mexico | 1779 | -100.1083 | 19.0439 |
| maiz_109 | onaveno | Sonora | 461 | -108.824 | 27.173 |
| maiz_110 | onaveno | Sonora | 626 | -109.68 | 29.809 |
| maiz_111 | RATON | Tamaulipas | 231 | -99.2551 | 24.1278 |
| maiz_113 | RATON | Nuevo Leon | 1001 | -100.7043 | 25.6813 |
| maiz_114 | reventador | Sonora | 204 | -108.912 | 26.844 |

| | | | | | |
|---|---|---|---|---|---|
| maiz_116 | tablilla de ocho | Chihuahua | 1791 | -106.632 | 28.32 |
| maiz_118 | TABLONCILLO | Sinaloa | 226 | -107.5619 | 25.4013 |
| maiz_119 | TABLONCILLO | Sinaloa | 113 | -105.6068 | 22.9493 |
| maiz_12 | Celaya | Guanajuato | 1877 | -101.1189 | 20.0911 |
| maiz_120 | TABLONCILLO | Sonora | 503 | -109.2448 | 27.827 |
| maiz_121 | TABLONCILLO | Sonora | 1570 | -108.9255 | 28.4106 |
| maiz_123 | TABLONCILLO_PERLA | Nayarit | 24 | -105.3903 | 21.991 |
| maiz_124 | tehua | Chiapas | 1545 | -93.206 | 17.216 |
| maiz_125 | tehua | Chiapas | 1525 | -93.175 | 17.216 |
| maiz_126 | TEPECINTLE | Chiapas | 2050 | -92.3193 | 15.3665 |
| maiz_127 | TUXPENO | Sinaloa | 86 | -106.1319 | 23.2539 |
| maiz_129 | TUXPENO | Nuevo Leon | 557 | -99.7227 | 24.7559 |
| maiz_131 | TUXPENO | Chiapas | 575 | -92.8193 | 16.0442 |
| maiz_132 | TUXPENO | Chiapas | 552 | -92.689 | 16.1194 |
| maiz_133 | tuxpeno_norteno | Mexico | 1390 | -100.228 | 18.66 |
| maiz_134 | tuxpeno_norteno | Nuevo Leon | 334 | -99.534 | 24.839 |
| maiz_135 | VANDENO | Sonora | 610 | -110.2122 | 29.796 |
| maiz_137 | VANDENO | Chiapas | 551 | -92.6964 | 16.1206 |
| maiz_138 | VANDENO | Chiapas | 612 | -92.9778 | 16.0422 |
| maiz_139 | ZAMORANO_AMARILLO | Jalisco | 450 | -104.6333 | 19.7167 |
| maiz_14 | Celaya | Guanajuato | 1823 | -101.4053 | 21.0086 |
| maiz_140 | ZAMORANO_AMARILLO | Jalisco | 300 | -104.45 | 19.6 |
| maiz_141 | ZAMORANO_AMARILLO | Michoacan | 1550 | -102.7167 | 19.9667 |
| maiz_142 | Zapalote_Chico | Chiapas | 654 | -93.002 | 16.364 |
| maiz_144 | Zapalote_Grande | Chiapas | 654 | -93.002 | 16.364 |
| maiz_146 | Arrocillo | Puebla | 2131 | -97.9803 | 19.9431 |
| maiz_148 | Celaya | Guanajuato | 1750 | -101.1611 | 20.3742 |
| maiz_149 | Chapalote | Sonora | 596 | -109.6743 | 29.8054 |
| maiz_150 | Comiteco | Chiapas | 1550 | -92.0201 | 16.2488 |
| maiz_151 | SERRANO_JALISCO | Jalisco | 2000 | -100.65 | 19 |
| maiz_152 | conejo | Guerrero | 1493 | -98.681 | 17.8856 |
| maiz_153 | conico | Hidalgo | 2143 | -98.405 | 20.2867 |
| maiz_154 | conico | Mexico | 2400 | -98.7817 | 19.0906 |
| maiz_156 | conico norteno | Chihuahua | 2019 | -106.6555 | 28.5187 |
| maiz_157 | coscomatepec | Puebla | 126 | -97.4557 | 20.1266 |
| maiz_159 | Dzit-Bacal | Chiapas | 779 | -93.4569 | 16.7518 |
| maiz_16 | elotero_sinaloa | Sinaloa | 220 | -105.5464 | 22.8897 |
| maiz_160 | conico | Mexico | 2600 | -98.7723 | 19.0556 |
| maiz_161 | conico | Tlaxcala | 2620 | -98.0472 | 19.3386 |
| maiz_162 | conico | Guanajuato | 2071 | -100.9356 | 20.7872 |
| maiz_163 | CRISTACH_GORDO_AZUL | Sonora | 1880 | -108.7097 | 29.8403 |
| maiz_164 | JALA | Nayarit | 1153 | -104.4287 | 21.1017 |

| | | | | | |
|---|---|---|---|---|---|
| maiz_165 | OLOTILLO | Chiapas | 191 | -92.2169 | 14.9018 |
| maiz_166 | OLOTON | Chiapas | 2299 | -92.5422 | 16.6398 |
| maiz_167 | OLOTON | Chiapas | 1220 | -92.1877 | 15.2961 |
| maiz_169 | JALA | Nayarit | 1057 | -104.44 | 21.0781 |
| maiz_17 | TEPECINTLE | Chiapas | 120 | -92.0889 | 17.3222 |
| maiz_170 | conico | Mexico | 2535 | -99.9294 | 19.7061 |
| maiz_171 | conico | Tlaxcala | 2584 | -98.3156 | 19.4875 |
| maiz_172 | conico | Hidalgo | 2081 | -98.3756 | 20.3547 |
| maiz_173 | elotero_sinaloa | Sinaloa | 258 | -106.4261 | 23.8461 |
| maiz_174 | elotero_sinaloa | Sinaloa | 1526 | -105.8308 | 23.4575 |
| maiz_176 | PEPITILLA | Mexico | 1779 | -100.1083 | 19.0439 |
| maiz_177 | TEPECINTLE | Chiapas | 100 | -92.1966 | 14.8203 |
| maiz_178 | TEPECINTLE | Chiapas | 899 | -92.4669 | 17.2507 |
| maiz_179 | conico | Mexico | 2663 | -99.9561 | 19.6511 |
| maiz_180 | conico | Hidalgo | 2297 | -98.2747 | 19.9986 |
| maiz_181 | conico | Tlaxcala | 2469 | -98.0694 | 19.3939 |
| maiz_182 | conejo | Guerrero | 1402 | -98.6564 | 17.7453 |
| maiz_183 | Comiteco | Chiapas | 1550 | -91.9375 | 16.1953 |
| maiz_184 | Chalqueno | Mexico | 2536 | -98.8039 | 19.1036 |
| maiz_185 | Arrocillo | Puebla | 2104 | -97.9794 | 19.9558 |
| maiz_186 | Comiteco | Chiapas | 1550 | -91.9749 | 16.2331 |
| maiz_187 | conico | Mexico | 2538 | -99.7911 | 19.5694 |
| maiz_188 | conico | Mexico | 2452 | -100.1675 | 19.352 |
| maiz_189 | conico | Hidalgo | 2104 | -98.3739 | 20.3589 |
| maiz_19 | SERRANO_JALISCO | Jalisco | 2160 | -103.6667 | 19.9333 |
| maiz_190 | conico | Mexico | 2576 | -100.0256 | 19.4908 |
| maiz_191 | conico | Mexico | 2560 | -98.8067 | 19.1086 |
| maiz_192 | conico | Mexico | 2479 | -98.6472 | 19.7594 |
| maiz_193 | VANDENO | Chiapas | 899 | -92.4669 | 17.2507 |
| maiz_195 | TABLONCILLO_PERLA | Nayarit | 54 | -105.1494 | 20.8742 |
| maiz_197 | Reventador | Sonora | 598 | -110.21 | 29.794 |
| maiz_198 | RATON | Tamaulipas | 199 | -99.0211 | 24.2901 |
| maiz_200 | OLOTILLO | Chiapas | 159 | -92.2045 | 14.8738 |
| maiz_201 | tablilla de ocho | Chihuahua | 1775 | -106.016 | 26.942 |
| maiz_202 | TABLONCILLO_PERLA | Nayarit | 20 | -105.2216 | 21.946 |
| maiz_21 | conejo | Guerrero | 1312 | -98.7431 | 17.7801 |
| maiz_23 | conejo | Guerrero | 1685 | -98.7419 | 17.778 |
| maiz_24 | conico norteno | Chihuahua | 2010 | -106.6323 | 28.4967 |
| maiz_25 | conico norteno | Chihuahua | 2010 | -106.6291 | 28.4949 |
| maiz_26 | coscomatepec | Veracruz | 201 | -96.7833 | 18.5 |
| maiz_27 | Ancho | Mexico | 2226 | -98.7786 | 18.9975 |
| maiz_28 | coscomatepec | Puebla | 1604 | -97.5649 | 19.9647 |
| maiz_29 | Ancho | Mexico | 2226 | -98.7786 | 18.9975 |
| maiz_3 | Apachito | Chihuahua | 1975 | -107.583 | 28.6858 |

| | | | | | |
|---|---|---|---|---|---|
| maiz_30 | Ancho | Mexico | 2073 | -98.8094 | 18.9681 |
| maiz_31 | Cacahuacintle | Mexico | 2585 | -99.5089 | 19.1964 |
| maiz_32 | Cacahuacintle | Mexico | 2700 | -99.6181 | 19.1675 |
| maiz_33 | cristalino de chichuahua | Chihuahua | 2094 | -106.8789 | 28.1932 |
| maiz_34 | cristalino de chichuahua | Chihuahua | 2034 | -107.4748 | 28.5028 |
| maiz_35 | Chalqueno | Mexico | 1390 | -100.2275 | 18.6603 |
| maiz_36 | Dulce | Chihuahua | 950 | -108.533 | 28.133 |
| maiz_37 | Chalqueno | Mexico | 2595 | -99.5953 | 19.4181 |
| maiz_39 | Dzit-Bacal | Chiapas | 618 | -92.9797 | 16.0323 |
| maiz_4 | Chapalote | Sonora | 537 | -109.2997 | 29.9044 |
| maiz_40 | Dzit-Bacal | Chiapas | 654 | -93.0019 | 16.364 |
| maiz_41 | elotes_occidentales | Guanajuato | 1800 | -100.7658 | 20.7442 |
| maiz_42 | elotes_occidentales | Guanajuato | 1854 | -100.8147 | 20.7844 |
| maiz_43 | conico | Mexico | 2585 | -99.5097 | 19.1847 |
| maiz_44 | conico | Mexico | 2605 | -100.0628 | 19.4606 |
| maiz_46 | JALA | Nayarit | 1060 | -104.4406 | 21.1 |
| maiz_47 | JALA | Nayarit | 1153 | -104.4287 | 21.1017 |
| maiz_49 | MUSHITO | Guanajuato | 1850 | -99.805 | 21.2775 |
| maiz_5 | Arrocillo | Puebla | 2159 | -97.9808 | 19.9341 |
| maiz_50 | MUSHITO | Guanajuato | 2500 | -100.1372 | 21.3575 |
| maiz_51 | NAL-TEL_TIERRA_FRIA | Oaxaca | 50 | -98.2167 | 16.3 |
| maiz_52 | NAL-TEL_TIERRA_FRIA | Oaxaca | 1250 | -96.7333 | 18.0167 |
| maiz_54 | Palomero | Chihuahua | 2769 | -106.44 | 26.36 |
| maiz_55 | conico | Mexico | 2576 | -100.0256 | 19.4908 |
| maiz_56 | conico | Mexico | 2538 | -99.7911 | 19.5694 |
| maiz_57 | conico | Distrito Federal | 2530 | -99.0503 | 19.1917 |
| maiz_58 | Palomero | Mexico | 2688 | -99.717 | 19.798 |
| maiz_6 | CRISTACH_GORDO_AZUL | Chihuahua | 2215 | -108.0152 | 28.4554 |
| maiz_60 | conico | Tlaxcala | 2358 | -97.6522 | 19.3192 |
| maiz_63 | Cacahuacintle | Tlaxcala | 2554 | -97.9008 | 19.2219 |
| maiz_64 | Cacahuacintle | Tlaxcala | 2745 | -98.0567 | 19.5997 |
| maiz_65 | conico | Tlaxcala | 2640 | -97.9247 | 19.1906 |
| maiz_66 | conico | Tlaxcala | 2621 | -97.9725 | 19.3217 |
| maiz_67 | Chalqueno | Tlaxcala | 2530 | -97.9881 | 19.3592 |
| maiz_68 | Chalqueno | Tlaxcala | 2497 | -97.9294 | 19.2908 |
| maiz_69 | Cacahuacintle | Hidalgo | 2133 | -98.4311 | 20.3392 |
| maiz_70 | Chalqueno | Hidalgo | 2068 | -98.3372 | 20.2214 |
| maiz_71 | Chalqueno | Hidalgo | 2136 | -98.2806 | 20.0297 |
| maiz_72 | conico | Hidalgo | 2059 | -98.3786 | 20.3317 |
| maiz_73 | conico | Hidalgo | 2144 | -98.2911 | 20.2433 |
| maiz_74 | conico | Hidalgo | 2104 | -98.3739 | 20.3589 |
| maiz_75 | conico | Hidalgo | 2081 | -98.3756 | 20.3547 |

| | | | | | |
|---|---|---|---|---|---|
| maiz_76 | OLOTILLO | Hidalgo | 225 | -98.4361 | 21.1333 |
| maiz_77 | OLOTILLO | Hidalgo | 194 | -98.3464 | 21.0639 |
| maiz_78 | OLOTILLO | Chiapas | 618 | -92.9797 | 16.0323 |
| maiz_79 | OLOTILLO | Chiapas | 654 | -93.0019 | 16.364 |
| maiz_8 | CRISTACH_GORDO_AZUL | Chihuahua | 2220 | -108.0158 | 28.4573 |
| maiz_80 | Comiteco | Chiapas | 455 | -93.0997 | 16.6231 |
| maiz_82 | Comiteco | Chiapas | 1553 | -91.977 | 16.1961 |
| maiz_83 | NAL-TEL_ALTURA | Chiapas | 1551 | -91.9769 | 16.2332 |
| maiz_84 | NAL-TEL_ALTURA | Chiapas | 1552 | -91.9164 | 16.2156 |
| maiz_85 | OLOTON | Chiapas | 2057 | -92.3145 | 15.3661 |
| maiz_86 | OLOTON | Chiapas | 1828 | -92.2936 | 15.3601 |
| maiz_88 | elotero_sinaloa | Sinaloa | 209 | -106.0817 | 23.4308 |
| maiz_89 | elotero_sinaloa | Sinaloa | 929 | -105.8917 | 23.4061 |
| maiz_91 | dulcillo del noroeste | Sonora | 1435 | -108.925 | 28.537 |
| maiz_92 | CRISTACH_GORDO_AZUL | Sonora | 1880 | -108.7097 | 29.8403 |
| maiz_93 | PEPITILLA | Mexico | 1830 | -100.1686 | 19.0525 |
| maiz_94 | PEPITILLA | Mexico | 1830 | -100.1686 | 19.0525 |
| maiz_97 | Arrocillo | Puebla | 2104 | -97.9794 | 19.9558 |
| maiz_99 | NAL-TEL_TIERRA_FRIA | Veracruz | 920 | -97.0667 | 18.85 |
